# The Role of RNA Editing in Cancer Development and Metabolic Disorders

**DOI:** 10.3389/fendo.2018.00762

**Published:** 2018-12-18

**Authors:** Che-Pei Kung, Leonard B. Maggi, Jason D. Weber

**Affiliations:** ^1^ICCE Institute, Washington University School of Medicine, Saint Louis, MO, United States; ^2^Division of Molecular Oncology, Department of Medicine, Washington University School of Medicine, Saint Louis, MO, United States; ^3^Siteman Cancer Center, Department of Cell Biology and Physiology, Washington University School of Medicine, Saint Louis, MO, United States

**Keywords:** RNA editing, ADAR, APOBEC1, cancer, metabolic disease

## Abstract

Numerous human diseases arise from alterations of genetic information, most notably DNA mutations. Thought to be merely the intermediate between DNA and protein, changes in RNA sequence were an afterthought until the discovery of RNA editing 30 years ago. RNA editing alters RNA sequence without altering the sequence or integrity of genomic DNA. The most common RNA editing events are A-to-I changes mediated by adenosine deaminase acting on RNA (ADAR), and C-to-U editing mediated by apolipoprotein B mRNA editing enzyme, catalytic polypeptide 1 (APOBEC1). Both A-to-I and C-to-U editing were first identified in the context of embryonic development and physiological homeostasis. The role of RNA editing in human disease has only recently started to be understood. In this review, the impact of RNA editing on the development of cancer and metabolic disorders will be examined. Distinctive functions of each RNA editase that regulate either A-to-I or C-to-U editing will be highlighted in addition to pointing out important regulatory mechanisms governing these processes. The potential of developing novel therapeutic approaches through intervention of RNA editing will be explored. As the role of RNA editing in human disease is elucidated, the clinical utility of RNA editing targeted therapies will be needed. This review aims to serve as a bridge of information between past findings and future directions of RNA editing in the context of cancer and metabolic disease.

## Introduction

Genetic complexity, or plasticity, is the foundation to develop complicated biological functions in living organisms. To maximize the versatility of limited amounts of genetic material, a variety of changes take place at the genomic level, including RNA metabolism and modification (1). RNA is involved in some of the most evolutionarily conserved cellular processes, including transcription and translation. The mechanisms of RNA regulation, including modification, processing and degradation, have been extensively studied. Among these mechanisms, site-specific substitution of RNA, or “RNA editing,” has garnered increasing attention in recent years, despite its discovery more than 30 years ago.

The year of 1987 marked the first milestone for the journey of RNA-editing. A cytidine (C) to uridine (U) conversion in the mRNA of human apolipoprotein B (apoB) was identified to be responsible for the production of a shorter version of apoB (apoB48) by creating a new stop codon (2). This alteration is mediated by an enzyme complex that contains the catalytic deaminase, apolipoprotein B mRNA editing enzyme, catalytic polypeptide 1 (APOBEC1) (3).

Meanwhile, a curious phenomenon of destabilization of double-stranded RNA was observed during the early embryogenesis of *Xenopus laevis* (4, 5). Hoping to use antisense RNA inhibition to study genetic factors in the embryonic development, investigators were surprised to learn that the same technique that works well in early-stage oocytes was not successful in later-stage oocytes and embryos due to failed formation of RNA duplex. This observation prompted speculations of a RNA-unwinding mechanism that either controls RNA stability or helps RNAs shape their secondary structures. It was further characterized by the loss of RNA's base-pairing properties and attributed to the conversion of adenosines (A) to inosines (I), an activity later found to be mediated by members of the adenosine deaminase acting on RNA (ADARs) family (6–8).

In the last 30 years, the physiological functions of APOBEC and ADAR protein family members have been gradually revealed (9, 10). These RNA editing enzymes (referred to as editases herein) can shuttle between the nucleus and cytoplasm, and homodimerization is required for their catalytic activity. APOBEC-mediated RNA editing has been implicated in maintaining homeostasis in digestive organs, such as the liver and small intestine, while ADAR-mediated RNA editing is thought to play a crucial role in regulating the innate immune response to infection. More recently, next generation sequencing has expedited the identification of specific RNA-editing targets and their associated functional consequences in human diseases.

**Figure 1:**
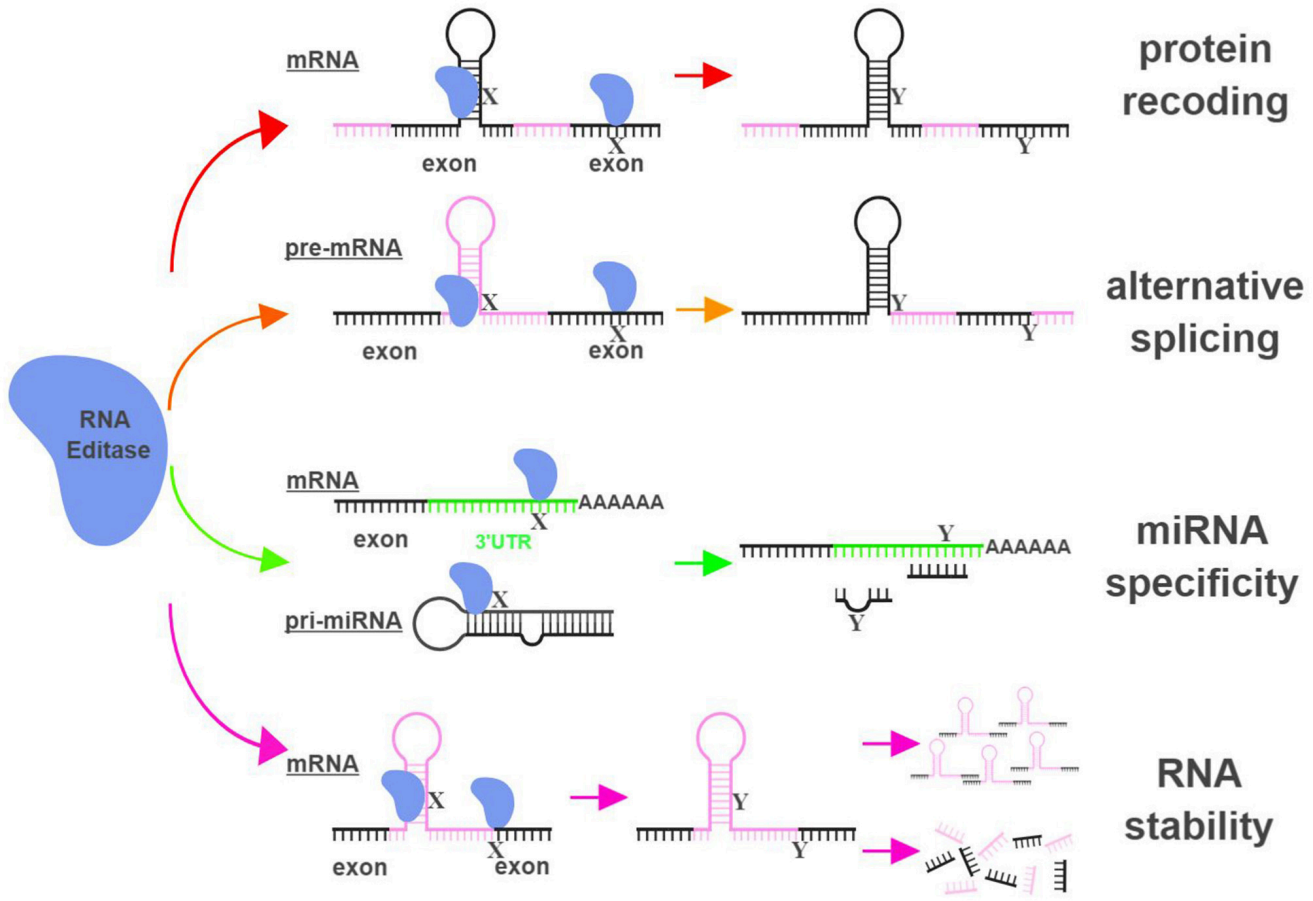
RNA editing leads to functional consequences through multiple mechanisms. RNA editases (ADAR1, ADAR2, ADAR3, and APOBECs in this review) regulate their targets through multiple mechanisms, including altering mRNA sequences in exons to change amino acid sequences (protein recoding; red arrows), changing splicing patterns of pre-mRNA to create novel products (alternative splicing; orange arrows), influencing miRNA specificity by altering seed sequences of miRNAs or sequences of miRNA targeting sites (miRNA specificity; green arrows), and directly impacting the stability of edited RNAs (RNA stability; magenta arrows). X represents a RNA base targeted by RNA editases (Adenosine for ADARs; Cytidine or Guanosine for APOBECs), and Y represents the resultant RNA base after the editing (Inosine for ADARs; Uridine or Adenosine for APOBECs). The hairpin structure in the mRNA represents Alu repeat elements that are frequently targeted by RNA editases. UTR, untranslated region. The figure was created with BioRender.

The functional impact of RNA editing on cell biology is demonstrated through (i) changing amino acid sequences of proteins (recoding); (ii) altering splicing patterns of pre-mRNA; (iii) causing changes in seed sequences of microRNAs (miRNAs) or in sequences of miRNA targeting sites; and (iv) influencing the stability of targeted RNAs (9, 10) (Figure 1).

This review aims to (1) provide a summary of recently identified RNA-editing events that regulate both cancer development and metabolic dysfunctions, (2) highlight the existing gaps in our knowledge of RNA-editing mechanisms, and (3) describe the potential implications for the development of novel therapeutic approaches to regulate RNA editing.

### RNA Editing in Cancer Development

Biogenesis of RNAs and RNA-regulated functions have been well-established in playing important roles in tumorigenesis (11). With the ability to change DNA-encoded genetic information after transcription, deregulated RNA-editing could be an important contributor in cancer development. Studies of RNA editing in a variety of cancer types (mostly in the context of A-to-I editing) have generated conflicting reports regarding the exact role RNA-editing plays.

The consistent finding from these reports is that RNA editing is a common phenomenon in cancer helping to drive transcriptomic and proteomic diversity, and overall levels of RNA editing mirror the expression levels of editases in cancers compared to normal tissues (ex. overexpression = general hyper-editing; reduced expression = general hypo-editing) (12–15). In contrast, the relationship between the overall editing level and tumorigenic potential of cancers appears to be unsettled. Increased level of RNA editing has been found to correlate with enhanced tumorigenesis in some cancers but reduced tumorigenesis in others, sometimes with both correlations in the same cancer type (12–14, 16–18).

These conflicting reports suggest that the relationship between RNA editing and cancer development is complicated and potentially influenced by other factors such as the origin, stage and microenvironment associated with the studied cancer. Instead of attempting to connect an individual cancer with the global level of RNA editing, connecting specific RNA editing events to cancer-related functions could prove to be more informative.

#### ADAR1

Currently, three *ADAR* gene family members have been identified and studied for their RNA-editing functions: ADAR1 (encoded by *ADAR*), ADAR2 (encoded by *ADARB1*) and ADAR3 (encoded by *ADARB2*) (9).

**Figure 2:**
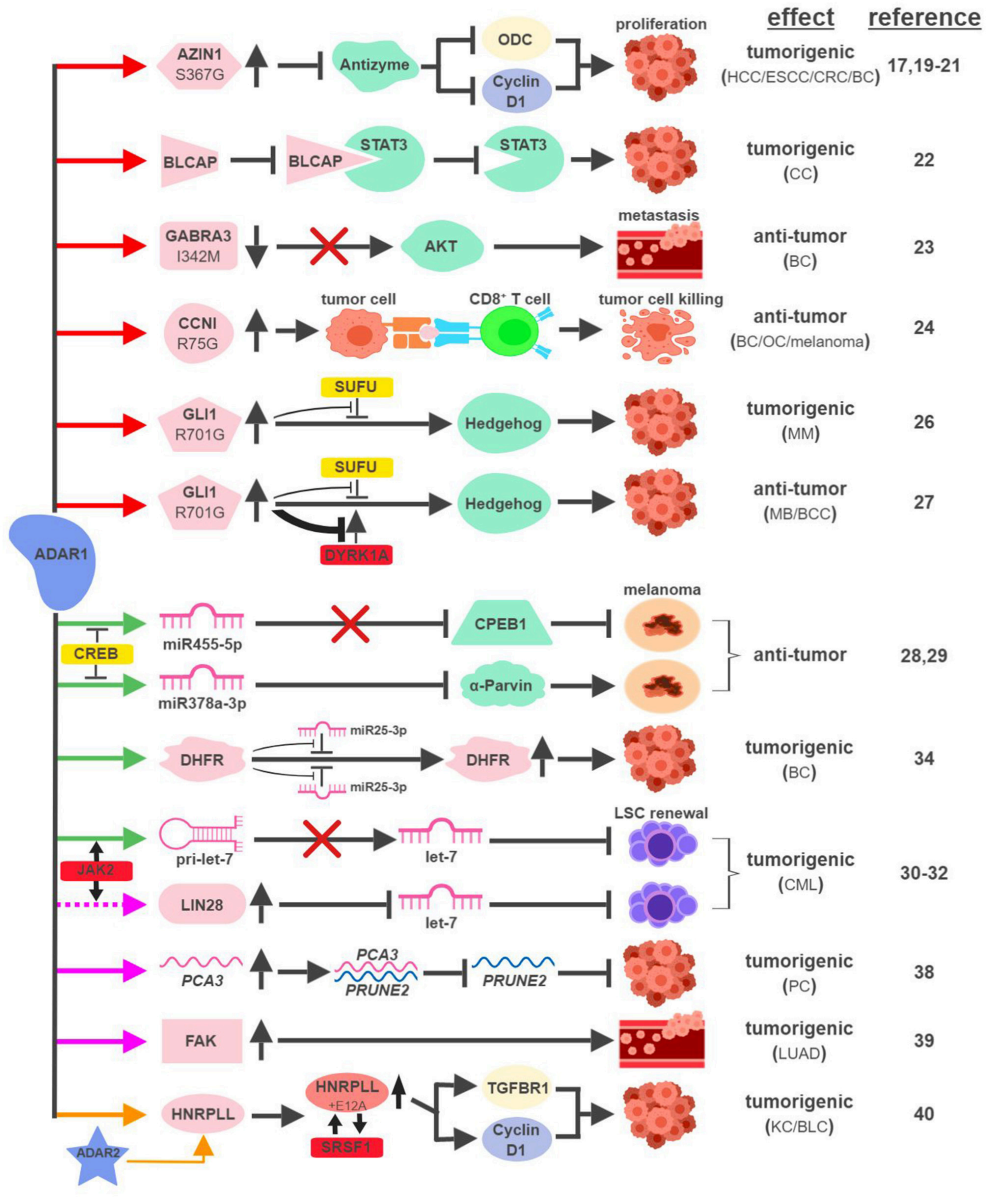
ADAR1-mediated RNA editing in cancer development. The color of the first arrow in each pathway indicates the mechanism (refer to Figure 1) by which ADAR1 regulates its direct targets, depicted in pink icons or shapes. Dashed lines indicate suggested/unproven functions/relationships. Additional activators and inhibitors of specific pathway steps are depicted in red and yellow rounded rectangles, respectively. Specific diseases and phenotypes/functions affected by ADAR1-mediated RNA editing are only labeled in the first appearance (ex. proliferation…etc). HCC, hepatocellular carcinoma; ESCC, esophageal squamous cell carcinoma; CRC, colorectal cancer; BC, breast cancer; CC, cervical cancer; OC, ovarian cancer; MM, multiple myeloma; MB, medulloblastoma; BCC, basal cell carcinoma; CML, chronic myeloid leukemia; PC, prostate cancer; LUAD, lung adenocarcinoma; KC, kidney cancer; BLC, bladder cancer. The figure was created with BioRender.

The mRNAs of multiple proteins have been identified as direct targets of ADAR1 and undergo nonsynonymous amino-acid substitutions associated with cancer development (Figure 2). In hepatocellular carcinoma (HCC), esophageal squamous cell carcinoma (ESCC), colorectal cancer (CRC) and breast cancer (BC), overexpression of ADAR1 leads to the creation of an oncogenic version of antizyme inhibitor 1 (AZIN1; S367G). Edited AZIN1 is stabilized and serves as an analog of ornithine decarboxylase (ODC) to block antizyme-mediated degradation of ODC and cyclin D1. Accumulations of ODC and cyclin D1 lead to increased cell proliferation and metastatic potential, as well as tumor initiating capacity (17, 19–21). In cervical cancer (CC), ADAR1 promotes tumorigenesis by editing multiple sites within the YXXQ motif of bladder cancer-associated protein (BLCAP), a tumor suppressor. Edited BLCAP loses its ability to interact with and inactivate signal transducer and activator of transcription 3 (STAT3), resulting in increased cell proliferation (22).

ADAR1 can exert anti-tumorigenic activities through RNA editing-mediated protein recoding, too. In BC, GABA_A_ receptor alpha 3 (GABRA3) activates the Akt pathway and promotes cell migration, invasion and metastasis. ADAR1-mediated editing of GABRA3 (I342M) reduces its cell surface expression and suppresses Akt activation and metastatic potential of cancer cells (23).

A recent study revealed a novel function of ADAR1 in cancer-associated immune environment. In subsets of tumor samples, including ovarian cancer (OC), melanoma and BC, increased levels of ADAR1-edited peptides are presented by human leukocyte antigen (HLA) molecules (24). Presentations of these edited peptides, such as cyclin I (CCNI; R75G), elicit antigen-specific killing of tumor cells through cytotoxic (CD8^+^) T cells. It presents an intriguing possibility to explore immunotherapeutic approaches utilizing information of RNA editing. Alternatively, it also poses a potential mechanism that cancers can hijack in order to avoid effective immune surveillance.

The boundary between pro-tumorigenic and anti-tumorigenic functions of ADAR1 can be blurry even with the same amino-acid-sequence-altering event. The glioma-associated oncogene 1 (GLI1) activates the Hedgehog (HH) signaling pathway to promote cell proliferation (25). In multiple myeloma (MM), amplified ADAR1 edits GLI1 (R701G) and stabilizes GLI1 expression by preventing the binding of its negative regulator, suppressor of fused (SUFU). Edited GLI1 displays higher transcriptional activity to drive HH signaling and promote malignant regeneration and drug resistance of MM (26). Interestingly, this exact same editing event has the opposite effect in medulloblastoma (MB) and basal cell carcinoma (BCC) to inhibit tumorigenesis. ADAR1-edited GLI1 (R701G), despite its resistance to SUFU binding, also becomes much less accessible to one of its activators, Dual specificity tyrosine-phosphorylation-regulated kinase 1A (DYRK1A). The net result is reduced oncogenic potential of edited GLI1 in MB and BCC (27).

In metastatic melanoma, ADAR1 confers tumor-suppressive activities by editing miRNA sequences to alter their target specificity. ADAR1-mediated editing of miR455-5p results in lack of inhibition of the tumor suppressor cytoplasmic polyadenylation element-binding protein 1 (CPEB1), while edited miR378a-3p targets the oncogene, α-Parvin, for downregulation (28, 29). During the course of melanoma progression, transcriptional repressors of ADAR1, such as cyclic AMP-responsive element binding protein (CREB), are upregulated to reduce ADAR1 expression to promote malignancy.

Not surprisingly, ADAR1 is also capable of hijacking the miRNA biogenesis process to promote tumorigenesis. In blast crisis chronic myeloid leukemia (BC CML), JAK2 activation and BCR-ABL1 amplification was shown to increase ADAR1 expression promoting leukemia stem cell (LSC) self-renewal (30). A follow-up study revealed that ADAR1 accomplishes this feat by reducing the expression of the tumor-suppressive miRNA let-7 (31). Mechanistically, overexpression of ADAR1 promotes the induction of the pluripotency gene *LIN28* and the editing of the primary miRNA pri-let-7 at multiple sites, both events reduce the production of let-7 family members (32, 33).

In addition to altering miRNAs directly, ADAR1 also edits miRNA targets to affect their susceptibility to miRNA-mediated repression. ADAR1 was shown to edit over two dozen sites on the three prime untranslated region (3'UTR) of dihydrofolate reductase (DHFR). In BC, edited DHFR becomes resistant to the targeting of miR25-3p and miR125a-3p. As a result, the protein levels of edited DHFR increase to promote cell growth and resistance to chemotherapeutic agents like methotrexate (34).

ADARs have been shown to regulate RNA stability through a variety of mechanisms, including alterations of subcellular localization or changing the secondary structure of edited RNAs (35–37). Although the detailed molecular processes remain elusive, several recent studies highlighted ADAR1's role in this capacity to impact cancer development. In prostate cancer (PC), ADAR1-mediated editing of *prostate cancer antigen 3* (*PCA3*), an intronic long noncoding RNA, increases its stability and expression. *PCA3* acts as a dominant-negative oncogene and forms a double-stranded RNA with precursor mRNA (pre-mRNA) of *prune homolog 2* (*PRUNE2*), a tumor suppressor gene (38). The formation of the *PCA3*-*PRUNE2* complex promotes tumorigenesis in cell and mouse models through the downregulation of PRUNE2, whose expression also inversely correlates with *PCA3* in human PC samples.

ADAR1-mediated RNA editing also occurs within the introns of protein-coding RNAs. One such example involves an important facilitator of tumor metastasis, focal adhesion kinase (FAK). In lung adenocarcinoma (LUAD), the most common form of non-small cell lung cancer (NSCLC), ADAR1-mediated editing of intron 26 of FAK results in increased stabilization of FAK mRNA and protein. Induction of FAK contributes to cell invasiveness and is associated with tumor recurrence in LUAD patients (39).

A recent study linking ADAR1 to intron-editing showed that ADAR1 edits the intron of heterogeneous nuclear ribonucleoprotein L-like (HNRPLL) to create an additional exon (E12A) for *HNRPLL* (40). E12A-containing *HNRPLL* acts as an enhancer of oncogenic splicing factor serine/arginine rich splicing factor 1 (SRSF1), resulting in a positive feed-back loop to increase the abundance of E12A-containing *HNRPLL* transcript. E12A-containing *HNRPLL* regulates expressions of cyclin D1 and transforming growth factor beta receptor 1 (TGFBR1) to promote cell proliferation in kidney and bladder cancers. Interestingly, this editing event is also mediated by ADAR2, pointing to potential interactions between ADAR1- and ADAR2-mediated RNA editing.

#### ADAR2

**Figure 3:**
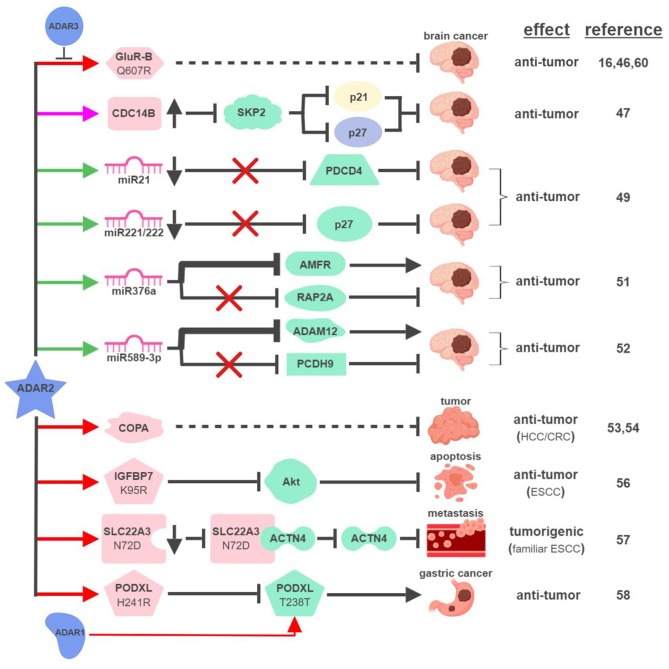
ADAR2-mediated RNA editing in cancer development. The color of the first arrow in each pathway indicates the mechanism (refer to Figure 1) by which ADAR2 regulates its direct targets, depicted in pink icons or shapes. Dashed lines indicate suggested/unproven functions/relationships. Specific diseases and phenotypes/functions affected by ADAR2-mediated RNA editing are only labeled in the first appearance (ex. brain cancer). HCC, hepatocellular carcinoma; ESCC, esophageal squamous cell carcinoma; CRC, colorectal cancer. The figure was created with BioRender.

First cloned in 1996, ADAR2 is the second identified A-to-I RNA editase that is also capable of editing itself (41, 42). ADAR2 was first identified as the main RNA editase of glutamate receptor subunit B (GluR-B) (Figure 3). Underediting of GluR-B (Q607R) results in early-onset epilepsy in a mouse model (43). Mice with mutant ADAR2 are also seizure-prone and experience early postnatal death, establishing the functional significance of ADAR2-mediated editing of GluR-B (44, 45). The translational impact of this connection was found in malignant human brain tumors in both adults and children, where ADAR2-mediated editing of GluR-B is reduced compared to control samples. Although brain tumors are not present in ADAR2-mutant mice, likely due to early postnatal death, these observations potentially explain the aggressive nature of these cancers and neurologic symptoms suffered by human patients (16, 46).

These early studies inspired mechanistic investigations to directly link ADAR2 with malignant brain tumors such as high-grade astrocytoma or glioblastoma multiforme (GBM). The same team that made the initial connection between ADAR2-mediated RNA editing and brain tumors identified several pathways downstream of ADAR2 regulating GBM pathogenesis. By editing multiple sites within intron 7 of the CDC14B phosphatase pre-mRNA, ADAR2 promotes upregulation of CDC14B, subsequently causing the degradation of E3-ligase S-phase kinase-associated protein 2 (SKP2) (47, 48). In GBM, downregulation of ADAR2-mediated RNA editing of CDC14B results in overexpression of SKP2. Increased level of SKP2 then leads to ubiquitin-mediated degradation of cell cycle inhibitors, p27^Kip1^ and CDKN1A/p21^Cip1/Waf1^, to promote cell cycle progression and tumorigenesis.

Interestingly, ADAR2-mediated downregulation of p27 is also connected to ADAR2's ability to edit selected miRNAs in the brain. ADAR2-mediated editing reduces expression of oncogenic miRNAs, such as miR21 and miR221/222. In GBM, failed editing/reduction of miR21 and miR221/222 lead to downregulation of their respective targets, tumor suppressors programmed cell death protein 4 (PDCD4) and p27^Kip1^ (49).

ADAR2 was found to edit numerous miRNAs, regulating tumorigenesis by balancing the functions of oncogenic and tumor-suppressive miRNAs (49, 50). This “balancing act” of ADAR2 can also be achieved by switching miRNAs between their oncogenic and tumor-suppressive activities via altering target specificities. The first such example was demonstrated by ADAR2's ability to edit miR376a to inhibit GBM progression (51). ADAR2-edited miR376a targets and downregulates autocrine motility factor receptor (AMFR) to inhibit tumor migration and invasion. Unedited miR376a, however, switches its affinity from AMFR to Ras-related protein RAP2A, a tumor suppressor protein acting on actin remodeling. In GBM, deactivated ADAR2 flips the switch through miR376a to tilt the balance toward malignant tumor progression.

A recent study revealed another ADAR2-controlled switch through miRNA editing in GBM. In normal brain tissues, ADAR2 edits nearly 100% of miR589-3p to target and reduce expression of disintegrin and metalloproteinase domain-containing protein 12 (ADAM12), a metalloprotease that promotes cancer metastasis. In high-grade GBM, editing of miR589-3p decreases dramatically and unedited miR589-3p targets protocadherin 9 (PCDH9), a tumor suppressor protein, instead (52). These studies likely represent only a small percentage of such “switches” regulating pathogenesis of GBM and other cancers.

In addition to GBM, ADAR2 also displays tumor-suppressive functions in other types of cancer. ADAR2 is downregulated in ~50% of HCC and overexpression of ADAR2 in ADARs-deficient HCC cells reduces their oncogenic potential (53). ADAR2's HCC-suppressing function is thought to be mediated through editing coatomer protein complex subunit α (COPA) mRNA (I164V), whose editing level inversely correlates with HCC pathogenesis. The lack of ADAR2-mediated editing of COPA was also observed in aggressive subtypes of CRC with an epithelial-to-mesenchymal (EMT) phenotype leading to liver metastasis (54).

Curiously, this seemingly straightforward assertion of “ADAR2 edits COPA to suppress tumorigenesis” is complicated by recent studies showing that (i) ADAR2 is overexpressed in a small subset of HCC (55); and (ii) edited COPA has been shown to promote a malignant phenotype of BC cells *in vitro* (15). This suggests that RNA-editing mechanism is subject to complex regulations to contribute to cancer-specific phenotypes.

ADAR2 can also serve as a dual-role regulator in esophageal cancers. In ESCC where ADAR2 is downregulated, reduced editing and expression of insulin-like growth factor-binding protein 7 (IGFBP7; K95R) lead to activation of Akt and inhibition of programmed cell death (56). In contrast, ADAR2 is a potential predisposing factor in familiar ESCC. Elevated levels of ADAR2 lead to editing and reduction of solute carrier family 22 member 3 (SLC22A3; N72D), which in turn promotes metastasis in familial ESCC by inhibiting the interaction between SLC22A3 and its inhibitory target α-actinin-4 (ACTN4), an actin-binding protein facilitating filopodia formation (57).

A recent study described an interesting “tug-of-war” relationship between ADAR2 and ADAR1 in gastric cancer (GC). Unedited podocalyxin-like protein (PODXL) promotes GC tumorigenesis by regulating cell adhesion mechanisms. PODXL can be targeted by both ADAR1 and ADAR2 to cause synonymous (ACA>ACG; T238T) and nonsynonymous (CAC>CGC; H241R) amino acid substitutions, respectively. In normal tissues, high ratio of ADAR2/ADAR1-mediated editing keeps PODXL in its edited form (H241R) preventing oncogenic onset. In GC, amplification of ADAR1 and reduction of ADAR2 cause imbalanced editing of PODXL to accumulate unedited PODXL to drive tumorigenesis (58).

#### ADAR3

Few studies have focused on investigating ADAR3's functional importance in RNA editing. ADAR3 shares significant sequence and structural similarities with ADAR1 and ADAR2, including nuclear localization, deaminase, and RNA-binding domains (59). Despite these similarities, ADAR3 has not been shown to display deaminase activity to influence physiological functions such as cancer development. A recent study demonstrated that ADAR3 may instead act as a dominant-negative form of other ADARs. Overexpressed in GBM compared to normal brain tissues, ADAR3 competes with ADAR2 for binding and editing of GluR-B through its RNA-binding domain (60). Given the observation that ADAR3 is expressed primarily in the brain, it is reasonable to speculate that ADAR3 regulates tumorigenesis of brain cancers by modulating ADAR2/ADAR1-mediated RNA editing (61).

#### APOBECs

In the human genome, there are eleven genes that belong to the APOBEC protein family. Functionally, APOBECs are cytidine deaminases and evolutionally conserved in vertebrates. The majority of research activities regarding APOBECs have focused on their ability to restrict viral infections by creating mutational imbalances in the viral genome (62). Recent developments, however, identified several APOBEC proteins, particularly APOBEC3 subfamily members (A3A, A3B, and A3H), being capable of catalyzing hypermutations in cancers to drive tumorigenesis and therapy resistance (63, 64). Interestingly, these connections have mostly been made with APOBECs' ability to modify single-stranded DNA instead of mRNA, despite what their names indicate (65).

**Figure 4:**
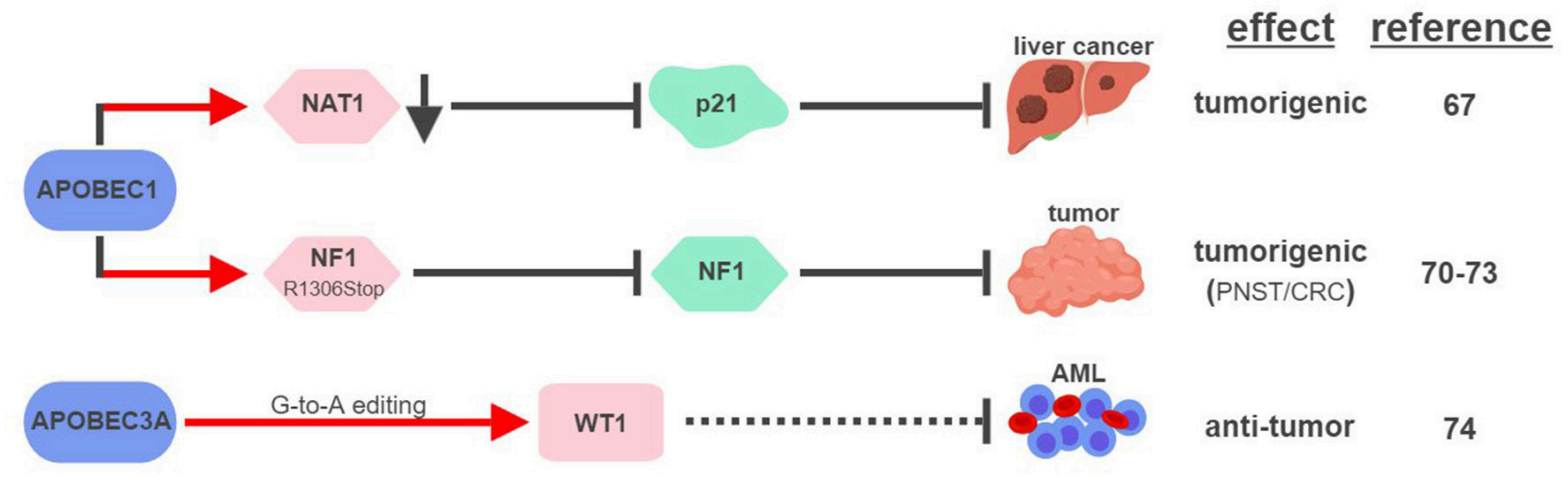
APOBECs-mediated RNA editing in cancer development. The color of the first arrow in each pathway indicates the mechanism (refer to Figure 1) by which APOBECs regulates its direct targets, depicted in pink shapes. Dashed lines indicate suggested/unproven functions/relationships. CRC, colorectal cancer; PNST, peripheral nerve-sheath tumor; AML, acute myeloid leukemia. The figure was created with BioRender.

So far, APOBEC1 is the most well-established APOBEC member that displays RNA editing activities (Figure 4). The first direct connection between APOBEC1-mediated RNA editing and tumorigenesis was made in 1995, when transgenic rabbits and mice expressing rabbit APOBEC1 in livers developed HCC (66). This outcome was found to be associated with hyperediting and reduced expression of a translational repressor NAT1 (novel APOBEC1 target no.1; also known as eukaryotic translation initiation factor 4 gamma 2, or EIF4G2), which regulates the expression of cell cycle inhibitor CDKN1A/p21^Cip1/Waf1^ (67, 68). APOBEC1 was also shown to bind the 3′UTR of *c-myc* mRNA to increase its stability (69). These results suggest that APOBEC1 might affect expression levels of tumor-associated genes via its RNA-binding and –editing capabilities.

Similar to its ability to edit *apoB* mRNA to create a truncated form apoB48 (Q2153Stop), APOBEC1 was found to edit the mRNA of neurofibromin 1 (NF1) to generate a truncated NF1 (R1306Stop) in a subset of peripheral nerve–sheath tumor (PNSTs) samples (70). It results in inhibition of the tumor-suppressor function of NF1, and could be responsible for development of neuronal tumors associated with Neurofibromatosis (NF) Type 1 (71, 72). Interestingly, increased expression of APOBEC1 and editing of NF1 were also found in CRC, suggesting that other tumor types could also be affected by this pathway (73).

Unconventional G-to-A RNA edits were identified in the mRNA of Wilms Tumor 1 (WT1) (74). These modifications increase in non-progenitor umbilical cord blood mononuclear cell samples (CBMCs) compared to acute myeloid leukemia (AML), implicating their roles in tumorigenesis. RNA interference screening identified APOBEC3A as the responsible RNA editase, opening up the possibility to investigate the relationships between all APOBEC members and cancer-associated RNA editing events. Interestingly, a functionally-important RNA conversion between C and U was also observed in WT1 in rat kidney during development (75). It is unclear which RNA editase is responsible for this conversion, but the fact that APOBEC1 is the only known C-to-U editase suggests that WT1 could be subject to RNA editing mediated by multiple APOBECs.

### RNA Editing in Metabolic Functions and Disorders

The fact that RNA editases (ADARs and APOBEC1) are expressed in major metabolic organs, such as the liver and pancreas, offers an initial clue that RNA-editing might play an important role in metabolic regulation (3, 76, 77). Deep-sequencing data collected longitudinally from one individual predisposed to and diagnosed with type 2 diabetes suggested that RNA-editing could have predictive and diagnostic values across healthy and diseased states (78). Moreover, RNA editing events in diabetes-associated genes were identified in human pancreatic islets from 89 deceased donors in an effort to uncover genetic mechanisms affecting glucose metabolism (79).

There is evidence to suggest that the RNA editing machinery is one of the evolutionarily-adaptive mechanisms developed while living organisms were becoming more complex. A-to-I RNA editing occurs mainly in primate-specific Alu repetitive elements that form secondary structures of dsRNA (80, 81). APOBEC1-mediated editing of apoB, thus the production of apoB48, only occurs in mammals (82). Evolutionarily-aligned genetic alterations are thought to have important metabolic consequences (83). Such speculation is prevalent when debating the origins of human metabolic diseases, hence the proposal of the thrifty gene hypothesis (84). Due to the lack of proper experimental models, few genetic events have been functionally proven to influence metabolic disorders in human. One recent example is the codon 72 polymorphism (Pro72Arg or P72R) in the tumor suppressor protein TP53 (85). The ancestral variant of this polymorphism (P72) is only present in primates, while the diversion from P72 to R72 only arose during the modern human evolution (86). Using cell and transgenic mouse models, the P72 variant was found to be a stronger responder to metabolic stresses to cause cell death, and the R72 variant is a predisposing factor for diet-induced obesity and diabetes (87, 88). Through its ability to regulate numerous target genes, RNA editing has potential to cause broader effects on human metabolic health than a single genetic alteration such as a mutation or a genetic polymorphism.

#### ADAR1

**Figure 5:**
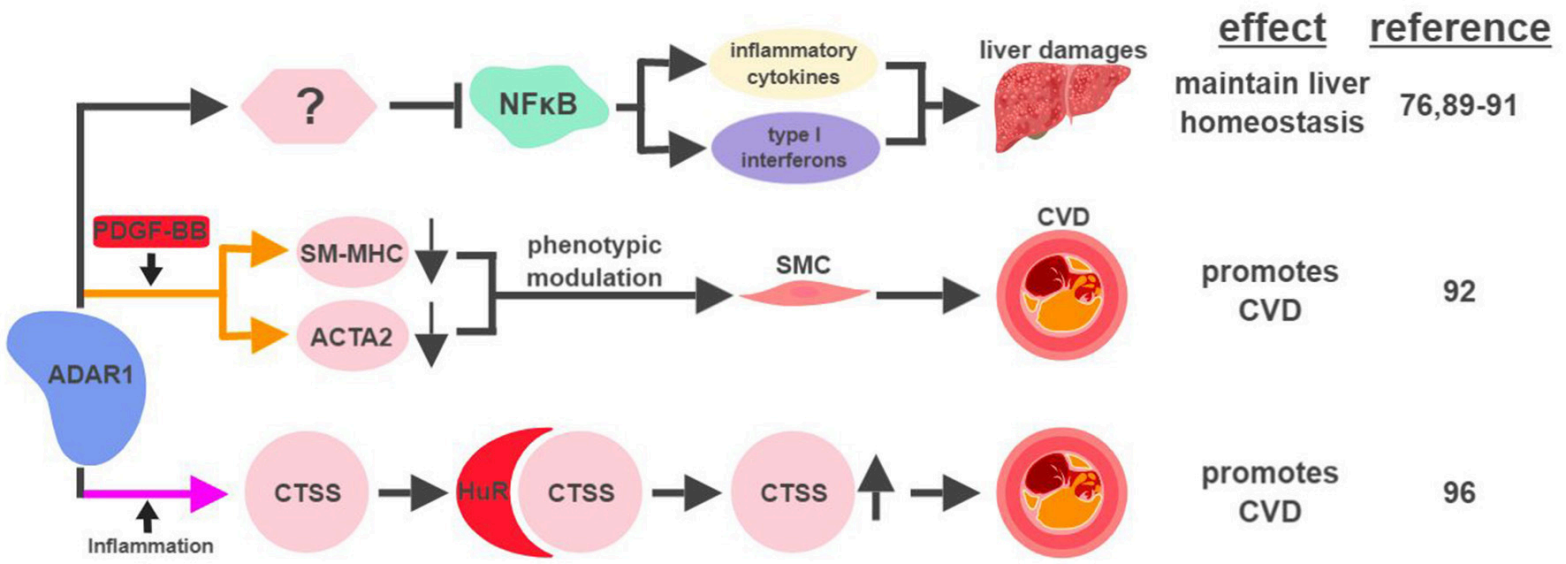
ADAR1-mediated RNA editing in metabolic disorders. The color of the first arrow in each pathway indicates the mechanism (refer to Figure 1) by which ADAR1 regulates its direct targets, depicted in pink shapes. Additional activators of specific pathway steps are depicted in red rounded rectangles. Specific diseases and phenotypes/functions affected by ADAR1-mediated RNA editing are only labeled in the first appearance (ex. CVD). CVD, cardiovascular disease; SMC, smooth muscle cell. The figure was created with BioRender.

It has been suggested that ADAR1 expression in the liver is important for early embryonic development. The absence of ADAR1 in the mouse liver results in impaired embryonic erythropoiesis, liver disintegration, and early death of the fetus (76, 89). Mechanistically, ADAR1 protects liver homeostasis by inhibiting inflammation (Figure 5). Silencing ADAR1 in liver cells induces levels of pro-inflammatory cytokines and type I interferons, partially through the NFκB pathway, to cause liver damage through inflammation, lipid accumulation, hepatitis and fibrosis (90, 91). ADAR1's ability to maintain liver homeostasis partially relies on its RNA-editing function, but no specific editing target has yet been identified as the responsible effector (90).

ADAR1-mediated RNA editing has recently been shown to contribute to cardiovascular disease (CVD). One mechanism for ADAR1 to promote CVD is through the phenotypic modulation of smooth muscle cells (SMC), a pivotal step during the development of CVD. The signature characteristic for the phenotypic modulation of SMC is the downregulation of SMC-specific genes, such as smooth muscle myosin heavy chain (SM-MHC) and smooth muscle α-actin (ACTA2), often mediated by platelet-derived growth factor (PDGF)-BB. In response to PDGF-BB, ADAR1 edits pre-mRNA of these SMC genes to cause abnormal splicing and subsequent downregulation of their mRNAs (92).

ADAR1's role to attenuate an aberrant innate immune response has been well established (93, 94). Its ability to inhibit unwanted inflammation also manifests in the form of Aicardi-Goutières syndrome (AGS), an autoimmune disease caused by ADAR1 mutations (95). Interestingly, ADAR1 acts as a promoting factor in the context of inflammation-driven CVD. Under hypoxia or pro-inflammatory conditions, ADAR1 expression is induced in endothelial cells to edit the 3′UTR of cathepsin S (CTSS) mRNA. This editing event promotes the recruitment of the RNA binding protein human antigen R (HuR) to stabilize the mRNA of CTSS, a cysteine protease known to be associated with atherosclerosis (96). Independent of its involvement in the regulation of the immune system, ADAR1 also plays critical roles regulating the development and homeostasis of multiple organs, including the spleen, small intestine, and kidney (97). These observations suggest that ADAR1 could impact metabolic functions in a systematic manner affecting multiple organs, possibly through its RNA-editing capability.

The links between circadian rhythms, sleep, and metabolism have been strengthened in recent years and presented as viable targets of therapeutic intervention for metabolic diseases (98, 99). A recent study, using *Drosophila melanogaster* as the model, demonstrated that deficiencies in ADAR1 results in synaptic dysfunction in glutamatergic neurons and sustained release of neurotransmitter to promote sleep (100). It underlines the variety of mechanisms ADAR1 could exploit to control an individual's susceptibility to metabolic disorders.

#### ADAR2

**Figure 6:**
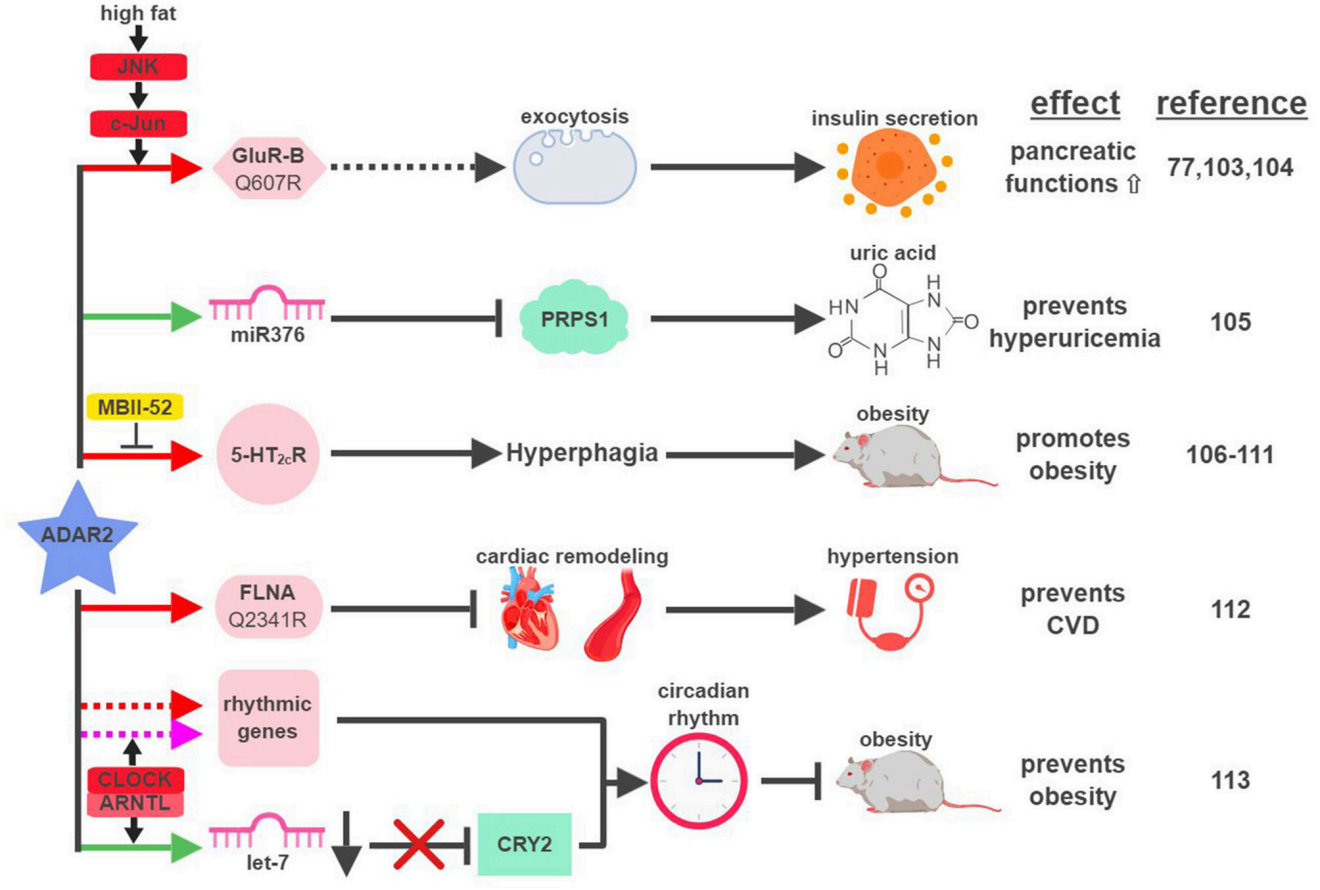
ADAR2-mediated RNA editing in metabolic disorders. The color of the first arrow in each pathway indicates the mechanism (refer to Figure 1) by which ADAR2 regulates its direct targets, depicted in pink icons or shapes. Dashed lines indicate suggested/unproven functions/relationships. Additional activators and inhibitors of specific pathway steps are depicted in red and yellow rounded rectangles, respectively. CVD, cardiovascular disease. The figure was created with BioRender.

The first identified target of ADAR2-mediated RNA editing is GluR-B, a subunit of the glutamate receptor (41). Around the same time of this discovery, glutamate receptors were found to regulate functions of pancreatic β-cells (101, 102). Despite this hint, a decade would pass before ADAR2 was directly connected to the metabolic functions of the pancreas (Figure 6). Using levels of ADAR2 expression and GluR-B editing as indicators in a mouse model, ADAR2 was found to be deactivated in pancreatic β-cells during fasting and activated in response to a high-fat diet (77). This regulation is mediated through the JNK-c-Jun pathway, as JNK-phosphorylated c-Jun acts as the transcription factor to induce ADAR2 expression in response to nutrient stimulation (103). Activated ADAR2 in turn promotes the secretion of insulin from the pancreas by influencing the expression of key factors involved in exocytosis (104). It remains to be seen if ADAR2-mediated editing of GluR-B is solely responsible for ADAR2's function in the pancreas, or if it involves other ADAR2 targets.

Certain metabolic diseases, such as diabetes or obesity, can manifest in the condition of hyperuricemia (abnormally high uric acid level). Interestingly, increased levels of uric acid were detected in the cortex of ADAR2-knockout mice (105). Hyperuricemia mediated by the loss of ADAR2 in the cortex correlates with the induction of phosphoribosyl pyrophosphate synthetase 1 (PRPS1), an essential enzyme involved in the synthesis of uric acid. Expression of PRPS1 is downregulated by miR376, whose seed sequence is edited by ADAR2 to increase its hybridization with PRPS1 mRNA.

Due to ADAR2's role in facilitating insulin secretion upon nutrient stimulation and reducing uric acid levels, one would assume that ADAR2 might be an active gate-keeper to prevent metabolic diseases. ADAR2-transgenic mice, however, develop hyperglycemia and severe obesity (106). ADAR2-induced obesity in transgenic mice is the result of altered behavior patterns presented in the form of addictive overeating (hyperphagia) (106, 107). Whether ADAR2-mediated RNA editing is necessary for this phenotype is unclear, as transgenic mice expressing mutant ADAR2 (E396A), defective for RNA-editing ability, developed similar levels of obesity compared to wild-type ADAR2 (106). On the contrary, strong evidence does exist to support the connection between ADAR2-mediated RNA editing and hyperphagia. For example, expression and editing levels of the ADAR2 target, serotonin 2C receptor (5-HT_2c_R), correlate with ADAR2 expression in the brains of ADAR2-transgenic mice and other mouse models of obesity (107, 108). In another study, transgenic mice solely expressing the fully-edited isoform of 5-HT_2c_R developed phenotypic characteristics of Prader-Willi syndrome (PWS), including hyperphagia (109). PWS is a genetic imprinting disorder that manifests in hyperphagia, early-onset obesity and diabetes. One of the imprinting genes lost from the paternal copies in PWS is the small nucleolar RNA (snoRNA) HBII-52 (MBII-52 in mouse). MBII-52 was found to specifically inhibit ADAR2-mediated RNA editing of 5-HT_2c_R, and loss of MBII-52 results in elevated 5-HT_2c_R RNA editing and PWS phenotypes (110, 111).

In CVD patients, RNA editing of Filamin A (FLNA), an actin crosslinking protein whose inactivation is linked to vascular abnormalities, is significantly reduced. ADAR2 was identified as the editase of FLNA, and ADAR2-edited FLNA (Q2341R) prevents cardiac remodeling and hypertension (112). This is the first known example linking ADAR2-mediated RNA editing to development of CVD through regulation of vascular function and blood pressure.

Like ADAR1, ADAR2 was also recently found to play a role in regulating circadian rhythm. CLOCK (Circadian Locomotor Output Cycles Kaput) – ARNTL (Aryl hydrocarbon Receptor Nuclear Translocator-Like protein (1) protein complex, a critical transcription factor during circadian cycles, was found to regulate the expression of ADAR2 and ADAR2-mediated RNA editing corresponding with the circadian rhythm in the liver (113). ADAR2 contributes to circadian clock maintenance through a couple mechanisms. First, ADAR2 regulates the recoding and stability of a subset of “rhythmic genes,” whose expressions align with the circadian cycle. Secondly, ADAR2 alters expression levels of major “clock proteins” (whose expression is essential for the circadian rhythmicity), such as Cryptochrome 2 (CRY2), by regulating biogenesis of their targeting miRNAs (in the case of CRY2, let-7) (114). ADAR2-knockout mice display disrupted rhythms of fatty acid metabolism and gain excessive weight with a high-fat diet, highlighting the significance of ADAR2 at the intersection between circadian rhythm and metabolic regulation.

#### ADAR3

Other than its preferred presence in the brain and inability to catalyze RNA editing on any proven target, little is known about ADAR3's functional connections to human diseases, including metabolic disorders. The aforementioned study in glioblastoma suggests a similar role for ADAR3 in metabolic diseases, as an inhibitor of RNA editing mediated by other active editases (60).

Despite the lack of mechanistic data, the large amount of genetic information available from the general population has shed some light on potential connections between ADAR3 and metabolism. Multiple single nucleotide polymorphisms (SNPs) in *ADARB2* (encodes ADAR3) were found to be associated with human longevity, using genetic information collected from centenarians in the US (115). Moreover, this association was later linked to a variety of metabolic parameters, including abdominal circumference, body mass index and serum triglyceride level (116). While the correlation between ADAR3 and aging was preliminarily demonstrated in mutant strains of *Caenorhabditis elegans*, more sophisticated models are needed to establish ADAR3's functional role in aging and metabolic regulation (115).

#### APOBECs

The first identified, and most studied, RNA editing target of APOBEC1 is apolipoprotein B (apoB; Q2153Stop) (2). Unedited and edited *apoB* encodes for a full-length form apoB100 and a truncated form apoB48, respectively. ApoB100 is synthesized in the liver and is a part of the assembly of low density lipoprotein (LDL) and very low density lipoprotein (VLDL), while apoB48 is mostly produced in the intestine and is required for chylomicron formation and fat absorption (117).

**Figure 7:**
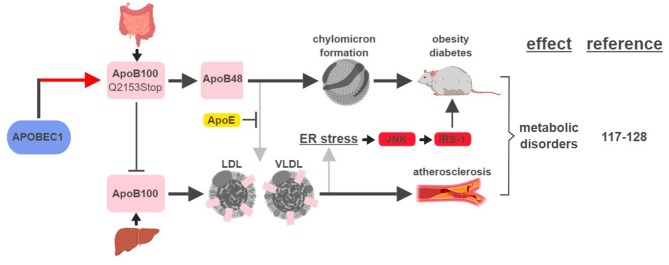
APOBEC1-mediated RNA editing in metabolic disorders. The color of the first arrow in each pathway indicates the mechanism (refer to Figure 1) by which APOBEC1 regulates its direct targets, depicted in pink shapes. Additional activators of specific pathway steps are depicted in red rounded rectangles. Gray arrows point to ApoB-associated secondary functions. ER, endoplasmic reticulum; LDL, low-density lipoprotein; VLDL, very-low-density lipoprotein. The figure was created with BioRender.

An elevated level of apoB100-containing LDL in the plasma is one of the major characteristics in patients with atherosclerosis, a disease state mimicked by mouse models that either lose APOBEC1's RNA editing activity or express apoB100 exclusively (118–120) (Figure 7). A recent genome-wide association study (GWAS) identified novel SNPs at *APOBEC1* that are associated with cholesterol composition, signifying APOBEC1's role in cholesterol-linked human diseases, such as atherosclerosis (121).

A transgenic rabbit model with reduced expression of APOBEC1 presented a lean phenotype compared to wild type when challenged with a high-fat diet (122). This phenotype is consistent with (i) apoB48's role in promoting chylomicron formation and lipid absorption, and (ii) observations in earlier studies that apoB48/apoB100 ratio and APOBEC1 expression were higher in obese and diabetic rats (123, 124).

However, the relationship between apoB and metabolic diseases is more complicated than just apoB48 (edited) leading to diabetes/obesity and apoB100 (unedited) leading to atherosclerosis. In an apoE-deficient mouse model, apoB48 promotes higher levels of cholesterol accumulation and atherosclerotic lesion formation. This phenotype is the manifestation of apoB48 being cleared exclusively through apoE, while apoB100 can be cleared through the LDL receptor alone (119, 125). On the other hand, links between apoB100 and obesity and diabetes have also been established. In rodent models fed high-fat diets, accumulation of apoB100 in the liver induces endoplasmic reticulum (ER) stress and insulin resistance (126, 127). This phenotype is caused by JNK-mediated phosphorylation of insulin receptor substrate (IRS-1), a connection known to link ER stress, obesity and diabetes (128).

These studies highlight the significant connections between apoB regulation, thus APOBEC1-mediated RNA editing, and metabolic disorders. Despite efforts made to identify other RNA editing targets of APOBEC1, no other target has yet been shown to play a role connecting APOBEC1-mediated RNA editing with metabolic disease (129). Data from one patient with a predisposition to type 2 diabetes showed that C-to-U editing is the second most frequent RNA editing event (next to A-to-I), indicating that RNA editing mediated by APOBEC1 or other C-to-U editases is an important factor in metabolic homeostasis (78).

### Gap in Knowledge and Future Directions

#### The Complexity Between RNA Editases

An established link of “Enzyme-Target-Function” provides the clearest blueprint to plan effective interventions of the RNA editing machinery. Numerous examples mentioned in this review fit this description providing multiple intervention points, including modulation of the levels and activity of editases, as well as correction of the edited target(s). There are, however, plenty of ambiguities in the world of RNA editing.

In the context of A-to-I editing, many disease-relevant editing targets lack clear identification of the responsible editase(s). Such examples include hyperediting-mediated alternative splicing of protein tyrosine phosphatase PTPN6 in AML, and hyperediting-activated ras homolog family member Q (RHOQ) in CRC (130, 131). In cases where involvement of editases were confirmed, the relationships among editases could be complicated. For example, ADAR1 and ADAR2, the two major A-to-I editases, can display either collaborative or antagonistic functions with each other. One example is the aforementioned editing of PODXL in GC. The disease outcome is not controlled by the function of one editase, but rather by the ratio between ADAR1- and ADAR2-mediated PODXL editing (58). More complications could come from ADAR3, whose role in RNA editing is just starting to be appreciated (60, 115). The possibility remains that further identifications and characterizations of proteins closely related to ADARs, such as ADAD1 (Adenosine Deaminase Domain Containing 1) and ADAD2, can further increase the complexity of A-to-I RNA editing (9).

The functional difference between the two ADAR1 isoforms, p150 and p110, is also an important factor to consider when determining ADAR1's role in human disease. Earlier studies characterized p110 as constitutively expressed, while p150 is interferon-inducible and the main isoform responsible for innate immune response modulation and AGS (93, 95, 132, 133). In the context of cancers and metabolic diseases, not all ADAR1-related studies have clearly differentiated the involvement between p150 and p110, creating potential issues to pinpoint the underlying mechanisms. As our understanding of functional distinctions between p150 and p110 improves overtime, there will be a need to revisit their individual roles in different diseases (97, 134–136).

As mentioned previously, APOBECs are better known for their abilities to edit DNAs in viral and tumor genomes. Recent identifications of APOBEC3A-mediated G-to-A RNA editing and APOBEC3G as a novel RNA editase signaled that (i) other APOBECs could also engage in RNA editing activities; and (ii) APOBEC-mediated RNA editing is not limited to the conversion from C to U (74, 137). In fact, APOBEC3A was recently shown to be a C-to-U RNA editase in immune cells, making it the first proven RNA editase capable of performing multiple RNA editing conversions (138). Further, albeit indirect, evidence to support these hypotheses is the recent realization that ADARs, well-known for their RNA editing functions, are also capable of performing DNA editing (139, 140). These developments spotlight not only the significance of carefully establishing the “Enzyme-Target-Function” connections, but also the untapped potential in uncovering the vast network of RNA editing in the context of human disease.

#### RNA Editing-Independent Functions

The effects between RNA and DNA editing can be distinguished through careful planning and execution of sequencing strategies. RNA editases, however, possess functions that are independent of their RNA-editing abilities. RNA editing-independent functions of ADARs were first noted in their effects on miRNA expression. Both ADAR1 and ADAR2 are capable of influencing miRNA expression by either directly interacting with miRNAs or affecting miRNA biogenesis through regulation of important factors, such as Dicer, Drosha or DGCR8 (DiGeorge Syndrome Critical Region 8) (141–143). RNA editing-independent functions of RNA editases play prominent roles in the processes of proliferation, metastasis, and immune evasion during tumorigenesis (49, 144–146).

The RNA editing-independent functions of ADARs have been demonstrated by utilizing their catalytically inactive forms. This approach has helped identify these non-catalytic functions beyond the confines of miRNA biogenesis. The MAPK-phosphorylated ADAR1 p110 isoform can be shuttled by Exportin-5 to the cytoplasm, where it protects the expression of anti-apoptotic genes by competitively inhibiting binding of Staufen1 to their 3′UTRs (134). In metastatic melanoma, RNA editing-incompetent ADAR1 is able to negatively regulate the expression of the metastatic enhancer integrin beta-3 (ITGB3) by (i) inhibiting the transcriptional activator of ITGB3, PAX6, and (ii) promoting FOXD1-mediated induction of miR22 to block ITGB3 translation (147). As mentioned previously, catalytically inactive ADAR2 mimics WT ADAR2 in an overexpression mouse model causing hyperphagia and obesity, dissociating this phenotype from ADAR2's RNA-editing capability (106).

Little information is available regarding RNA editing-independent functions of C-to-U editases, such as APOBEC1. However, APOBEC-mediated functions that don't require its deaminase domains have been reported in humans and other species (148, 149). As more functional studies of RNA editases are reported, clear differentiations between RNA editing-dependent and –independent mechanisms will be necessary to adequately assess their contributions to the development of cancers and metabolic diseases.

#### Regulations of RNA Editing

RNA-editing events are subjected to highly precise regulatory mechanisms. Mechanisms that regulate general localization and expression of RNA editases have been well-studied (9, 10). Functional regulation of RNA editing in the context of cancer and metabolic disease, however, remains a gap in our knowledge. Depending on the tissue of origin and disease stage, different cancers have been associated with overall induction or reduction of RNA-editing levels (12, 13, 18). Even in diseases with either a clear overall editing profile (hyper- vs. hypo-editing) or an apparent alteration of RNA editase expression, many targets are edited in the opposite manner (12). Moreover, alterations of a single editase do not always yield the same result, as demonstrated by the aforementioned pro- and anti-tumorigenic functions displayed by both ADAR1 and ADAR2 in different cancers.

More dramatic examples can be found in situations where the same editing event leads to completely different functional or phenotypic outcomes. Such examples include ADAR1-mediated editing of GLI1 and ADAR2-mediated editing of COPA in tumorigenesis (15, 26, 27, 53). APOBEC1-produced apoB48 and its full-length counterpart apoB100 contribute to developments of atherosclerosis and obesity to different extents based on the surrounding regulatory environment (119, 125). Even replicating a complex editing profile on one target, such as editing of 5-HT_2c_R, could result in opposite phenotypes (obesed vs. lean) in two different animal models (109, 150).

Several regulatory mechanisms of RNA editing have been recently identified. In aggressive forms of BC, such as triple-negative BC and metaplastic BC, the presence of ADAR1 is important for their tumorigenic capacity. Recent studies found that in these cancers, the expression and activity of ADAR1 can be regulated by tumor-promoting proteins CPSF6 (cleavage and polyadenylation factor-6) and mutant RPL39 (ribosomal protein L39, A14V). CPSF6 interacts with ADAR1 to stabilize its localization and enhance its RNA editing activity (151). Moreover, CPSF6-mediated activation of ADAR1 can be inhibited by prolactin, a mammary differentiation factor. The oncogenic mutant of RPL39 (A14V) induces expression of ADAR1 to promote tumor growth and chemoresistance through the functions of iNOS (inducible nitric oxide synthase) and activated STAT3 (152).

In the brain, where functions of ADAR2 have been extensively studied, a splicing factor SRSF9 (serine and arginine rich splicing factor 9) was found to repress ADAR2-mediated RNA editing. SRSF9 interacts with ADAR2 and its editing targets in the nucleus to disrupt the formation of ADAR2 dimer, which is necessary for the editing of genes involved in controlling cell survival (153, 154). These findings also signaled the importance of the splicing machinery in the regulation of RNA editing (155). In CRC, PKCζ (protein kinase C zeta) phosphorylates ADAR2 to activate its RNA editing activity. Phosphorylated ADAR2 inhibits liver metastasis of CRC by promoting the accumulation of miR-200, potentially through editing of COPA and other targets (54).

Considering the complex relationship between ADAR1- and ADAR2-mediated RNA editing, it is not surprising that mechanisms exist to regulate their RNA-editing functions simultaneously. One recent example of this is the RNA helicase, DHX9 (DEAH box helicase 9). By using an overexpression system in an esophagus carcinoma cell line (EC109), DHX9 was found to preferentially promote and repress ADAR1- and ADAR2-mediated RNA editing, respectively (156). The end result is a strong correlation between DHX9 expression and tumorigenesis. High-throughput screening has been employed to identify endogenous regulators of ADAR-mediated RNA editing (157, 158). Attempts to identify additional enhancers and inhibitors, both intrinsic and extrinsic, of the RNA editing machinery are ongoing.

APOBEC1-mediated C-to-U RNA editing is carried out in a multiprotein “editosome” (159). Many components that are important for the function of this editosome have been identified, including ACF (APOBEC1 complementation factor), HNRNPAB (heterogeneous nuclear ribonucleoprotein A/B; or ABBP-1), DNAJB11 (DnaJ heat shock protein family member B11; or ABBP-2), KSRP (KH-type splicing regulatory protein), CELF2 (CUGBP Elav-like family member 2; or CUGBP2), SYNCRIP (synaptotagmin binding cytoplasmic RNA interacting protein; or GRYRBP), and RBM47 (RNA binding motif protein 47) (160–166). A rare negative regulator of this editosome, BAG4 (Bcl2-associated Athanogene-4), was found to suppress APOBEC1-mediated RNA editing by shuttling APOBEC1 to the cytoplasm (167). Aside from ACF and RBM47, the physiological significance of these regulators remains to be confirmed beyond *in vitro* experiments (166, 168).

Although more investigations are needed to confirm the roles of these APOBEC1 partners in APOBEC1-regulated cancer development, an interesting study using a mouse model of testicular germ cell tumors (TGCTs) hinted strongly at such connections. Using 129/Sv inbred mice that develop spontaneous TGCTs, *Apobec1* deficiency was found to affect TGCT susceptibility either alone or in combination with mutations of *Dnd1* (Deadend1), another TGCT risk factor that shares strong sequence homology with Apobec1-editosome member Acf (169). Pending validation of the involvement of Apobec1-mediated RNA editing in this model, this result suggests that APOBEC1-mediated impact on tumorigenesis is subjected to complex regulatory mechanisms, possibly involving one or more members of the editosome. More interestingly, the effect of Apobec1 deficiency on TGCT susceptibility was influenced by the context of germ-lineage (maternal vs. paternal) and it manifests in a transgenerational manner. It suggests that APOBEC1 regulates heritable epigenetic changes, presumably through RNA-editing, to impact the development of human diseases such as testicular cancer (170).

One major regulator of metabolism, peroxisome proliferator-activated receptor alpha (PPARα), impacts APOBEC1-mediated RNA editing *in vivo*. In mice lacking LDL receptor, PPARα agonist ciprofibrate (a common cholesterol-lowering drug) reduces hepatic RNA editing of apoB by decreasing the expression of Acf (171). The result is increased accumulation of apoB100-associated VLDL and atherosclerosis. It also demonstrated that regulatory mechanisms of RNA editing could impact human diseases by affecting their response to treatments.

Since RNA editing plays a prominent role in cancer development, tumorigenesis-associated pathways and factors could prove to be important regulators of the machinery. For example, tumor suppressor protein TP53 is involved in nearly all aspects of tumorigenesis, but few connections have been made between TP53 and RNA editing (172). So far, TP53 has only been deleted to create viable cell models to study ADAR1-mediated RNA editing in the innate immune response (93). The fact that TP53 status influences the ADAR1-associated phenotype indicates a larger role for TP53 in the world of RNA editing. A recent study identified IFI16 (interferon gamma inducible protein 16) as a common interacting partner of ADAR1 and p53, strengthening this possibility (173).

There are stronger hints for roles of another TP53-related protein, ARF (alternative reading frame; or CDKN2A/p14), in RNA editing. ARF is well known for its ability to activate TP53 by inhibiting MDM2 (mouse double minute 2), but many TP53-independnet functions of ARF have also been identified (174). One of such functions was recently demonstrated in triple-negative BCs, where ARF collaborates with TP53 to suppress a tumor-promoting inflammatory pathway involving interferon-β and STAT1, which are also important factors in ADAR-mediated RNA editing (90, 93, 132, 175). Combined with the fact that both ARF and ADAR use the nucleolus as a critical hub, it seems to place ARF in close proximity to the center of RNA editing universe (176–178).

As our understanding of RNA editing in cancers and metabolic disorders improves, it is likely that many other connections will be discovered between RNA editing and established factors associated with these human diseases.

### Opportunities for Therapeutic Applications

With greater understanding of the relationship between RNA editing and human disease comes the opportunity for innovative therapeutic approaches. RNA-based therapies, which include targeting both RNA itself and its modifications, are becoming viable options to slow down or even reverse the course of human disease (179–181). This warrants further investigation of the mechanisms of RNA-editing and including it as an integral part of RNA-based therapies. Indeed, not only the overall role of RNA editing is being studied in various diseases, but also creative molecular technologies are being developed to identify and verify specific RNA editing events using cell lines or clinical samples (12, 17, 182). High-throughput sequencing and “omics” profiling enable researchers to create comprehensive “maps” of RNA editing in the human transcriptome (158, 183, 184). A recent study integrated genomic, transcriptomic, and proteomic data to pinpoint RNA editing events that are directly responsible for proteomic diversity leading to disease-relevant alterations in cancer samples (15).

#### Modulation of RNA Editing

In principle, modulating the expression or activity of RNA editases is a reasonable strategy for treating diseases driven by dysregulated RNA editing. In cancer cells with elevated ADAR-mediated RNA editing, such as breast and lung cancers, downregulation of ADAR expression reduces their tumorigenic capacity (17, 39, 185). To counteract apoB48-mediated chylomicron formation and lipid absorption, expression of APOBEC1 was reduced to create transgenic rabbits that are resistant to diet-induced obesity (122). Beyond altering the expression of RNA editases, molecular tools are also being developed to perform selective inhibition of RNA editing. For example, target-specific inhibition of RNA editing has been demonstrated by using either morpholino-based or 2′-O-methyl/locked nucleic acid mixmer antisense oligonucleotides (186, 187).

In situations where the RNA editing level is inversely correlated with disease progression, promoting RNA editing could have beneficial effects. In cancer cells where ADAR2 is downregulated, overexpression of ADAR2 displays tumor-suppressive activity (16, 53). Overexpression of APOBEC1 combined with an endothelial functional modulator, SR-BI (scavenger receptor, class B, type I), was tested in a cell culture model to show anti-atherogenic potential by altering lipoprotein composition and increasing nitric oxide levels (188). To augment the effect of RNA editing, artificial and manipulatable tools have been engineered to control the RNA editing machinery. SNAP-tag technology was used to assemble, through covalent bonding, a RNA-editing complex containing the catalytic domain of ADAR1 and a guide RNA. By integrating a light-sensitive protection molecule between the editase and the guide RNA, the target–specific RNA editing machinery can be switched on and off via light (189).

#### Application of RNA Editing

The concept of creating a RNA-guided editase has been adopted to attempt target-specific RNA editing. Proof-of-principle studies have been conducted to demonstrate the potential to target disease-relevant genes and restore proper protein function through RNA editing (190, 191). This strategy has also been applied through the CRISPR (Clustered Regularly Interspaced Short Palindromic Repeats)-Cas genomic editing system. By fusing the deaminase domain of ADAR2 with a catalytically inactive Cas13, this complex can be led by a guide RNA to perform specific and robust RNA editing (192, 193). Continuous efforts to increase the efficiency, reduce off-target editing, and promote simultaneous editing of multiple targets, will push these technologies closer to therapeutic application (194, 195).

#### Unintended Consequences and Unique Opportunities

As intriguing as the idea to reverse disease conditions by modulating RNA editing levels, it is not without potential drawbacks. Since different human diseases are associated with either elevated or reduced levels of RNA editing, altering it one way or the other poses the risk of undesired consequences. For example, it is theoretically possible to modulate the overall levels of inosine-containing RNA by regulating ribonuclease V (196, 197). But dysregulation of ribonuclease V has been linked to cancers and psychiatric disorders, indicating the potential hazards (198, 199). This concern can be extended to approaches targeting an individual RNA editase, as mutations of ADAR1 and ADAR2 have been linked to devastating genetic diseases (95, 200).

In addition to impacting disease progression, RNA editing could also affect drug response. In MM, ADAR1-mediated RNA editing promotes immunomodulatory drug resistance (26). APOBEC1-mediated RNA editing of apoB influences the liver's response to lipid-lowering drugs like fibrates (171). Moreover, ADAR1 can directly alter the cellular response to a drug by regulating the RNA editing and expression of xenobiotic-metabolizing-related factors, such as AhR (aryl hydrocarbon receptor) and CYP1A1 (Cytochrome P450, family 1, member A1) (201). These studies highlight potential side-effects of both stand-alone and combinatorial therapies involving modulation of RNA editing.

Man-made, RNA-guided, and target-specific RNA editing has the potential to become the next revolution in (epi)genetic therapy. It offers a unique opportunity to modulate protein functions without altering the sequence and integrity of the genome. Indeed, its unique characteristics haven't gone unnoticed and RNA editing has been incorporated into cutting-edge technologies, such as CRISPR-Cas genome editing (192). Recent studies pointing out the potentially crippling effects of genome editing, however, should serve as a cautionary tale when considering using RNA editing in a similar fashion (202–205). Further studies are needed to ensure the efficacy and safety of this approach before considering clinical applications.

Having mentioned the potential problems, it should be acknowledged that there are tremendous opportunities for RNA-editing-based therapies to treat human diseases. By engaging biological events at the RNA level, RNA-editing could be associated with many-to-one or one-to-many relationships between RNA editases/events and downstream effects. Examples of “many-to-one” include the “see-saw” effect of PODXL editing by ADAR1 and ADAR2 in GC, and the ability of both ADAR1 and ADAR2 to regulate biogenesis of miRNA let-7 (31, 49, 58). It points out common downstream effectors of multiple editases, offering therapeutic targets that are more specifically linked to diseases. There are also “one-to-many” cases, such as the association of ADAR2-mediated editing of miR376 with both glioblastoma and metabolic disorders (51, 105). Intervention strategies targeting “ADAR2-miR376” as a unit thus could have broader range of applications.

The relationship between cancer development and metabolic disorders has been strengthened in recent years, and it appears to be a two-way street. It is well-established that cancers often overcome unwanted stresses by hijacking metabolic pathways, and metabolic disorders like obesity and diabetes are strong predisposing factors to cancers (206, 207). Reversely, cancers can create systematic metabolic imbalances in patients resulting in metabolic diseases such as diabetes or CVD (208, 209). Understanding the cross-talk between cancer and metabolic diseases is one of the most critical challenges for human health, and RNA editing is an important piece of the puzzle.

## Conclusion

As this review shows, there has been an explosion of information regarding RNA editing in the last 3–5 years. This is indeed an exciting time to study this unique epigenetic phenomenon, with plenty of opportunities and challenges ahead. Some burning questions, as highlighted throughout this review, will need to be answered in the near future. How is new information used to reshape the central dogma of cell biology of “DNA to RNA to protein?” Are there more RNA editases waiting to be discovered? How do we reliably identify RNA editases, and corresponding editing events, in specific human diseases? Can part of the RNA editing machinery be targeted as a monotherapy, or is combining these interventions with other parallel treatments, such as immunotherapy, a better course of action?

Outside the purview of this article, RNA editing also plays significant roles in other physiological conditions, such as infectious, inflammatory/autoimmune, and neurodegenerative diseases (210–213). It will be interesting, in some cases necessary, to investigate the inter- and intra-relationships between the roles of RNA editing in these diseases with those in cancers and metabolic disorders. The ultimate goal is to leverage this information into actionable therapeutic innovations. More than 30 years after its initial discovery, the significance of RNA editing in human disease is being recognized more than ever.

## Author Contributions

CK and JW conceived of the topic of the manuscript. CK drafted the manuscript and created images with BioRender. CK, LM, and JW revised the manuscript.

### Conflict of Interest Statement

The authors declare that the research was conducted in the absence of any commercial or financial relationships that could be construed as a potential conflict of interest.
